# Maternal Transfer of the Cyanobacterial Neurotoxin β-N-Methylamino-L-Alanine (BMAA) via Milk to Suckling Offspring

**DOI:** 10.1371/journal.pone.0078133

**Published:** 2013-10-23

**Authors:** Marie Andersson, Oskar Karlsson, Ulrika Bergström, Eva B. Brittebo, Ingvar Brandt

**Affiliations:** 1 Department of Environmental Toxicology, Uppsala University, Uppsala, Sweden; 2 Department of Pharmaceutical Biosciences, Uppsala University, Uppsala, Sweden; Wadsworth Center, New York State Dept. Health, United States of America

## Abstract

The cyanobacterial neurotoxin β-N-methylamino-L-alanine (BMAA) has been implicated in the etiology of neurodegenerative disease and proposed to be biomagnified in terrestrial and aquatic food chains. We have previously shown that the neonatal period in rats, which in humans corresponds to the last trimester of pregnancy and the first few years of age, is a particularly sensitive period for exposure to BMAA. The present study aimed to examine the secretion of ^14^C-labeled L- and D-BMAA into milk in lactating mice and the subsequent transfer of BMAA into the developing brain. The results suggest that secretion into milk is an important elimination pathway of BMAA in lactating mothers and an efficient exposure route predominantly for L-BMAA but also for D-BMAA in suckling mice. Following secretion of [^14^C]L-BMAA into milk, the levels of [^14^C]L-BMAA in the brains of the suckling neonatal mice significantly exceeded the levels in the maternal brains. In vitro studies using the mouse mammary epithelial HC11 cell line confirmed a more efficient influx and efflux of L-BMAA than of D-BMAA in cells, suggesting enantiomer-selective transport. Competition experiments with other amino acids and a low sodium dependency of the influx suggests that the amino acid transporters LAT1 and LAT2 are involved in the transport of L-BMAA into milk. Given the persistent neurodevelopmental toxicity following injection of L-BMAA to neonatal rodent pups, the current results highlight the need to determine whether BMAA is enriched mother's and cow's milk.

## Introduction

The neurotoxic amino acid β-N-methylamino-L-alanine (BMAA) was early implicated in the etiology of the Amyotrophic lateral sclerosis/Parkinsonism-dementia complex (ALS/PDC) on the island of Guam [1], and more recently an association of BMAA to ALS and Alzheimer's disease in North America has been proposed [2]–[4]. The evidence for the presence of BMAA in human brain tissue is however equivocal and the potential role of BMAA in neurodegenerative disease remains controversial [5].

The cyanobacterial toxin BMAA was originally reported to be produced by symbiotic cyanobacteria living in roots of cycad trees and found to be present in cycad seed flour as well as in the tissues of flying foxes feeding on these fruits [6], [7]. Based on these studies, it was for the first time demonstrated that cyanobacterial BMAA is transported in a food chain, resulting in high exposures of indigenous people consuming cycad flour and flying foxes at the island of Guam [8], [9].

Seasonal mass growths of cyanobacteria are known to produce a variety of toxins affecting the brain, liver and other tissues. The cyanobacteria are however ubiquitous organisms found in a variety of aquatic and terrestrial environments [10], [11]. Recently, BMAA has been detected in several water systems, including temperate aquatic ecosystems, and in mollusks and fish suggesting that BMAA can bioaccumulate also in aquatic food chains [12], [13]. Studying the presence of BMAA in marine biota in southern Florida, Brand et al. [12] reported high levels in shellfish and in various fish species used for human consumption. High BMAA levels have also been found in shark fins, blue crabs and in feathers of flamingos collected at an African lake [14]–[16]. Resulting from eutrophication, cyanobacterial blooming have increased in the Baltic Sea, and BMAA was recently identified in muscle and brain tissue of various species of fish and in mussels collected in Baltic waters [13]. Altogether, these recent results strengthen the hypothesis that BMAA can biomagnify in food webs. The magnitude of human exposure to BMAA may consequently be higher than previously anticipated, and several unforeseen BMAA sources need to be considered to elucidate the extent of human exposure to BMAA.

The rate of BMAA transfer into the adult rodent brain is low [17]–[19] and BMAA is reported to have a relatively low neurotoxic potency in adult rodents [20]. However, in rodent fetuses and neonates the uptake of BMAA in discrete brain regions is more efficient and selective than in adults [18], [21]. Accordingly, early life-stage exposure to BMAA affects the development of the brain in rodents with long-term consequences also in adulthood. For instance, subcutaneous exposure of rodent pups to BMAA during lactation results in dose dependent alteration in striatal neuropeptides involved in neuronal survival, as well as in long-term cognitive and proteomic impairments and neurodegeneration observed when the exposed rodents have reached adult age [22]–[25].

The neonatal model (post-natal day (PND) 9–10) used in our previous studies is the first animal model that shows significant biochemical and behavioral effects after BMAA short-term exposure at a dose of 40 mg/kg [23], [25], [26]. The time period PND 9–10 is an important phase of brain development in rodents with rapid maturation of neuronal systems and there is often an increased susceptibility to neurotoxicants during this period [27], [28]. The corresponding period in humans starts during the last trimester of pregnancy and continues through the first few years of age [27]. The oral bioavailability of BMAA has been shown to be almost complete in both rats and primates [29], [30]. Human exposure to BMAA may occur via contaminated drinking water supplies or following food-chain transfer and fetuses could possibly be exposed to BMAA following transplacental transport as previously demonstrated in pregnant mice [18]. The potential exposure via secretion of BMAA to mother's milk is presently unknown.

The aim of the present study was to examine and compare the secretion of the two enantiomers of BMAA, ^14^C-labeled L- and D-BMAA, into milk in lactating mice. We also examined the gastrointestinal absorption of the enantiomers in the suckling pups and the subsequent transfer of the BMAA into the developing brain. To gain some information on the transport kinetics in the milk-producing cells, we employed the murine mammary epithelial HC11 cell line to examine the influx and efflux of BMAA following differentiation into a secretory phenotype. The results suggest that secretion into milk is an important elimination pathway for BMAA in lactating mothers. Accordingly there was a high exposure of nursed offspring to BMAA during a sensitive period of neurodevelopment.

## Materials and Methods

### Chemicals

The HCl salt of β-N-methylamino-L-alanine [methyl-^14^C] ([^14^C]L-BMAA) and β-N-methylamino-D-alanine [methyl-^14^C] ([^14^C]D-BMAA) were purchased from Onco Targeting AB, Uppsala, Sweden. Specific radioactivities were 57 mCi/mmol for both enantiomers and radiochemical purities were 99%. Other chemicals were purchased from Sigma-Aldrich Co., St Louis, MO, USA if not stated otherwise.

### Animals and housing

Pregnant C57BL/6 mice arrived on gestational day 15 from Taconic, Ejby, Denmark. They were housed alone in standard macrolon cages with wooden chip bedding, paper houses and paper towels as nesting material. The animals were maintained at 22°C with a 12 h light/dark cycle and they were given standard pellet food (R36 Labfor; Lantmännen, Kimstad, Sweden) and tap water *ad libitum*. One day postpartum the litter sizes where normalized to 9 pups/dam.

All animal experiments were approved by the Uppsala animal ethical committee (permit number: C 233/11) and followed the guidelines of Swedish legislation on animal experimentation (Animal Welfare Act SFS1998:56) and European Union legislation (Convention ETS123 and Directive 86/609/EEC).

### Administration of [^14^C]BMAA

On postnatal day 9 the lactating dams where divided into two groups, four (D-BMAA) and five (L-BMAA) dams/group. The mothers were separated from their pups 30 min prior to administration and kept separated for another 30 min after administration in order for the pups to grow hungry and for the substance to enter the mammary gland. The dams were injected intravenously with either [^14^C]L-BMAA or [^14^C]D-BMAA, 250 µCi/kg body weight (0.7 mg/kg body weight), diluted in Hanks balanced salt solution (HBSS). In addition, one spare dam having only four pups was given [^14^C]L-BMAA as above.

### Autoradiographic imaging

At 3, 8 and 24 h after returning to their respective lactating dam, one pup from each litter was removed and euthanized by exposure to gaseous CO_2_. The pups were immediately embedded in an aqueous gel of carboxyl methyl cellulose (2.5% w/v) and water, and frozen in hexane cooled with solid CO_2_. The frozen blocks were mounted in a large cryostat microtome and sagittal sections (20 µm) through different levels of the body were collected onto tape, as described by Ullberg [31]. A total of 15-20 tissue-sections representing all major tissues and fluids in the body were collected from each animal. At termination of the experiment after 24 h, one dam per exposure group was killed and sectioned as described above. The four pups from the spare dam were removed at 2 h. For comparative purposes, this dam with discontinued nursing was also killed at 24 h and sectioned as described above. After freeze-drying, all tissue-sections were apposed to X-ray film for autoradiographic exposure. The tissue-sections were apposed to film for 200 days (dams) and 240 days (pups).

### Sampling of milk and brain

To determine the levels of radioactivity in milk and brain tissue, two pups/dam were taken at the time points 1, 3, 8 and 24 h from five and four dams in the [^14^C]L-BMAA and [^14^C]D-BMAA exposed groups respectively. The pups were euthanized by decapitation and the coagulated stomach milk and the brains were dissolved in Soluene®350 (PerkinElmer). Radioactivity was measured using a TriCarb scintillation counter (PerkinElmer) after addition of Ultima Gold™ scintillation cocktail. Data was calculated as cpm/mg tissue or stomach milk, respectively.

### In vitro studies using HC11 cells

#### Cell culture

The mouse mammary epithelial cell line HC11 was a kind gift from Dr. B. Groner [32]. The cells were grown in RPMI1640 culture medium with 2 mM L-glutamine and NaHCO_3_ complemented with 10% fetal bovine serum, 5 µg/ml insulin, 0.01 µg/ml EGF, 100 units of penicillin and 100 µg/ml streptomycin. They were differentiated into a lactating phenotype according to a protocol described by Jäger [33] with some modifications of the length of differentiation period. In short, confluent cells were made competent to receive lactogenic stimuli by culturing them in EGF-deficient medium for 48 h and then differentiated by changing into culture medium with 100 nM dexamethasone, 5 µg/ml insulin and 5 µg/ml prolactin for 4 days.

### Uptake and time course studies

HC11 cells, grown and differentiated in 24-well plates, were incubated in pre-heated (37°C) Hank's Balanced Salt Solution, HBSS, containing 1 µM [^14^C]BMAA (0.5 ml/well) for 1, 10, 20, 30, 40 and 60 min. Uptake of [^14^C]BMAA into the cells was stopped by addition of ice-cold PBS (>1 ml/well) and the cells were subsequently washed four times with >1 ml ice-cold PBS. Cells were lysed with 0.5 ml 1 M NaOH and 0.3 ml was transferred to a scintillation vial. Following addition of 5 ml Ultima Gold scintillation cocktail the radioactive content was measured in a TriCarb liquid scintillation counter. The protein concentration in the cell lysates was determined using Pierce ™ BCA protein assay. The results from the time course studies served as a ground for the time points chosen for the following studies.

### Efflux studies

In the efflux studies the cells were pre-loaded with 1 µM [^14^C]L- or [^14^C]D-BMAA for 40 min at 37°C and then quickly washed once with an excessive amount of PBS (room temperature). Fresh, pre-heated HBSS-medium was then added (1 ml/well). To measure the rate of efflux of labeled BMAA from the cells, 0.5 ml medium was removed at 5, 10, 20, 30, 40 and 80 min and radioactivity was measured after addition of Ultima Gold Scintillation cocktail. At the same time-points, efflux was stopped by wash of cells with ice-cold PBS. The cells were then lysed and analyzed for radioactive content as described above.

### Concentration-dependent uptake studies

In order to determine the saturation kinetics of L-BMAA, cells were exposed to increasing levels of unlabeled L-BMAA spiked with ^14^C-labeled BMAA (0.3–5 mM). The exposure was stopped after 15 min with ice-cold PBS and radioactive content of the cells was analyzed as described above. *K*_m_ and *V*_max_ values were determined by fitting the data to the Michaelis-Menten equation.

### Temperature and sodium dependency

Facilitated transport over the cell membrane is a temperature dependent process and both influx and efflux of [^14^C]BMAA were studied at 4°C. Loading of cells with [^14^C]BMAA prior to efflux at 4°C was performed at 37°C for 40 min. To investigate sodium-dependency, a choline substituted uptake study was performed using a standard Kreb-Ringer's buffer with or without sodium ions, i.e sodium ions had been replaced during the preparation step with choline chloride (120 mM) [34]. Exposure was stopped after 15 min with ice-cold PBS and radioactive contents of the cells was measured as described above.

### Competition with other amino acids

The influx of [^14^C]L-BMAA was also determined in the presence of the amino acids phenylalanine, alanine and serine. Phenylalanine is known to be transported by the sodium-independent neutral amino acid transporters LAT1 and LAT2, while alanine and serine are transported by a range of amino acid transporters including ASCT and LAT2 [35], [36]. Unlike LAT1 and LAT2, ASCT is a sodium-dependent transporter [35], [37]. The cells were pre-incubated with pre-heated HBSS containing the competing amino acid (0.2 µM-10 mM) for 15 min at 37°C before changing to 1 µM [^14^C]L-BMAA with the same concentrations of the competing amino acid. Exposure was stopped after 15 min with ice-cold PBS and radioactive content of the cells was measured as described above.

### Viability assay

In order to determine that the flux of BMAA over cell membrane was not influenced by unspecific toxicity of BMAA, a typical uptake study with increasing concentrations of L-BMAA (0.3 µM– 1.25 mM; 15 min, 37°C) was performed with the addition of the tetrazolium dye MTT to the wells, 0.5 mg/ml, and further incubation at 37°C for 3 h. Viable cells with functioning mitochondria will reduce MTT to insoluble formazan [38]. At the end of the 3 h incubation period the exposure medium was removed without disrupting the cells. 0.5 ml of 0.08 M HCl in isopropanol was added to the wells and cells were lysed and formazan dissolved at 37°C for 15 min. Absorbance was measured at 560 nm with a background absorbance at 750 nm.

### Statistical analyses

The in vivo experiment was conducted with five dams for [^14^C]L-BMAA exposure group and four dams for [^14^C]D-BMAA exposure group and two pups were taken per time point. The statistical unit was considered to be the dam and the mean concentration of radioactivity recorded in the two pups sampled at each time-point was therefore used for the statistical analysis. The results were expressed as mean ± SD. The in vitro experiments were run at least three times, at different days using different passages, and the studies were performed using triplicates or more. To determine the statistical significance between groups, a nonparametric Mann-Whitney *U*-test was applied and a statistical difference was considered at *p*<0.05. The Graph Pad software Prism 5 was used for the statistical analyses.

## Results

### Autoradiographic imaging of [^14^C]L- and [^14^C]D-BMAA in lactating mice

**Figure 1:**
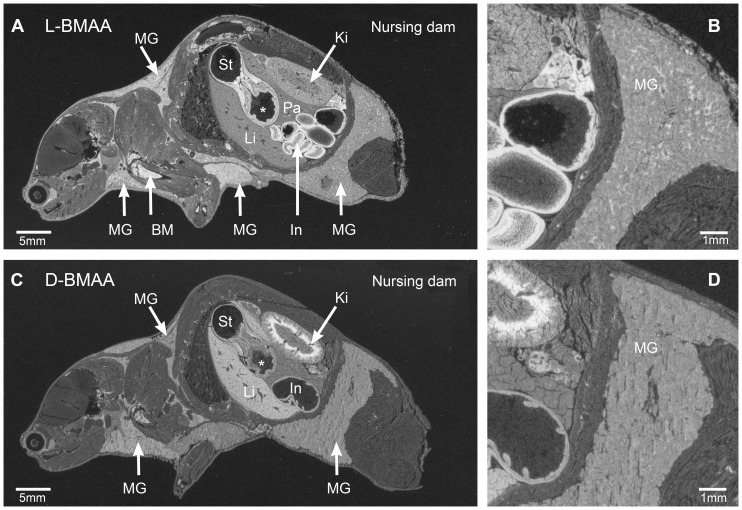
Tissue localization of [^14^C]-labeled L- and D-BMAA in nursing mouse dams. Autoradiograms showing high levels of radioactivity in the mammary glands (MG) of nursing dams 24 h after injection of [^14^C]L-BMAA (**A**, **B**) or [^14^C]D-BMAA (**C**, **D**). Enlargements of the posterior mammary glands are shown in **B** and **D**. White areas correspond to high levels of radioactivity. BM  =  bone marrow, St  =  stomach, *  =  glandular part of stomach, In  =  lower gastrointestinal tract, Ki  =  kidney, Li  =  liver.

**Figure 2:**
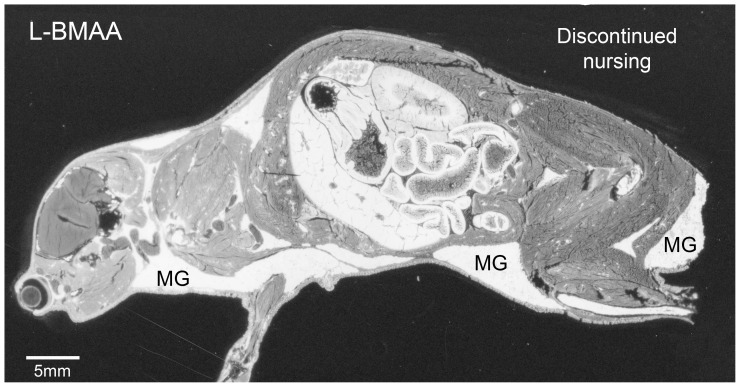
Tissue localization of [^14^C]-labeled L-BMAA in a lactating mouse dam with discontinued nursing. Autoradiogram showing very high levels of radioactivity in the mammary glands (MG) and other tissues of a lactating dam that nursed for 2 h and was killed 24 h after injection of [^14^C]L-BMAA. Compare the level of radioactivity of the dam nursing for 24 h (**Figure 1A**). The high retention of labeled substance in the mammary glands and all other tissues in the dam with discontinued nursing is due to interruption of milk excretion. White areas correspond to high levels of radioactivity.

All adult mice subjected to autoradiography were killed at 24 h after a single intravenous injection of ^14^C-labeled BMAA and continuous nursing of suckling pups. Results were interpreted with the assumption that the major part of radioactivity in milk and brain tissues was due mainly to unchanged BMAA, and not to metabolites. The general biodistribution patterns of [^14^C]L-BMAA and [^14^C]D-BMAA in lactating mice are shown in Figure 1A and 1C. The overall retention of [^14^C]L-BMAA in the lactating mouse with the all the suckling pups removed after 2 h is shown in Figure 2. Considerably higher levels of radioactivity remained in the tissues of the [^14^C]L-BMAA exposed lactating mouse with discontinued nursing than in the tissues of the nursing mice exposed either to [^14^C]L- or [^14^C]D-BMAA. The general level of [^14^C]L-BMAA was considerably higher in the dam with discontinued nursing, particularly in the mammary glands, than in the mice with completed nursing during the investigative period. Overall distribution pattern was, however, similar in all mice exposed to [^14^C]L-BMAA (Fig. 1A and 2). In nursing mice given either [^14^C]L- or [^14^C]D-BMAA, the levels of radioactivity in the milk ducts, i.e. milk, were in general higher than in the mammary parenchymal tissue (Fig. 1B). The level of both labeled enantiomers in blood was very low and below that of most other tissues. [^14^C]L-BMAA gave rise to a high uptake of radioactivity in the epithelia of the gastrointestinal tract, particularly in the small and large intestinal mucosa, the bone marrow but also in lymphatic tissues such as the thymus and spleen (white pulp), i.e. in tissues with a high protein synthesis and cell turnover. The uptake of [^14^C]L-BMAA in the adult brain and spinal cord was fairly low and homogenously distributed. The uptake in the liver and pancreas was intermediate. A distinct uptake in the outer layer of the lens was also evident as well as in the Harderian gland adjacent to the eye.

The biodistribution of [^14^C]D-BMAA was roughly similar to that of [^14^C]L-BMAA, although the uptake in the intestinal mucosa, bone marrow and in thymus and spleen was less pronounced (Fig. 1C). While [^14^C]L-BMAA, was evenly distributed in the kidney, [^14^C]D-BMAA gave rise to a selective accumulation of radioactivity in the inner part of the kidney cortex (Figs. 1C and 1D). However, a similar phenomenon can be seen in the [^14^C]L-BMAA exposed dam with discontinued nursing and [^3^H]L-BMAA exposed mice in an earlier study [21].

### Autoradiographic imaging of [^14^C]L- and [^14^C]D-BMAA in suckling pups

**Figure 3:**
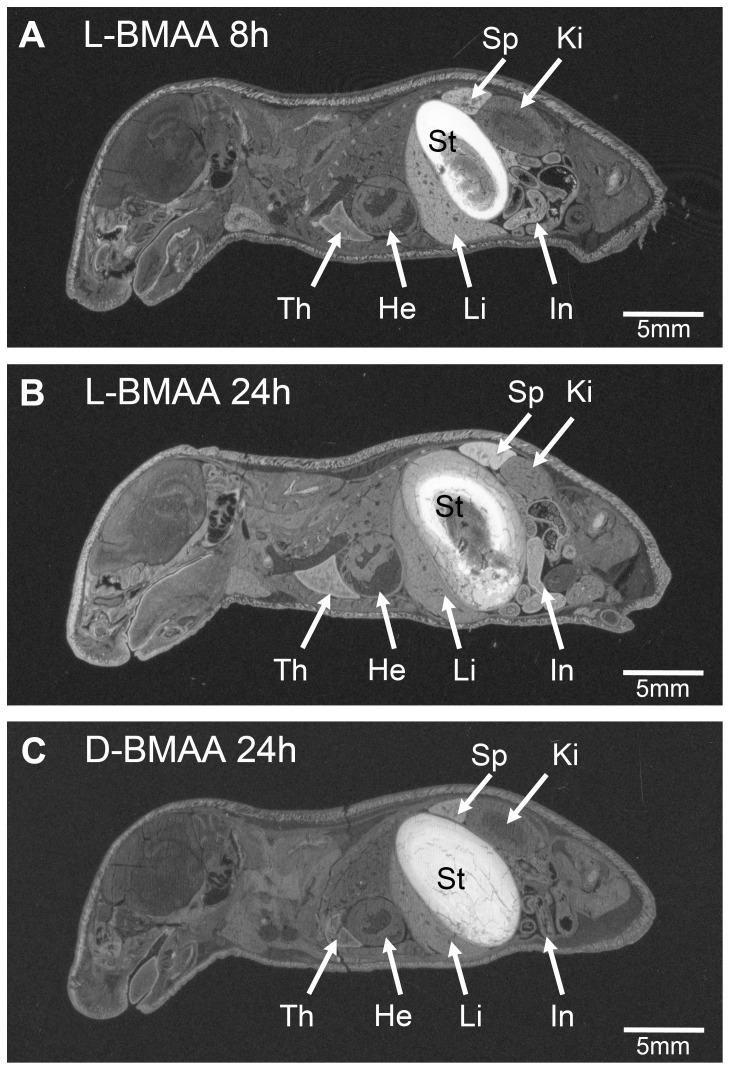
Tissue localization of [^14^C]-labeled L- and D-BMAA in suckling pups following a single administration to the nursing dams. Autoradiograms showing very high levels of [^14^C]L- and [^14^C]D-BMAA enantiomers in coagulated milk filling the stomach (St) of pups nursed for 8 or 24 h. White areas correspond to high levels of radioactivity. Th  =  thymus, He  =  heart, St  =  stomach, In  =  lower gastrointestinal tract, Ki  =  kidney, Li  =  liver, Sp  =  spleen.

High levels of radioactivity were present in the contents of the stomach (coagulated milk) of the suckling pups killed after 3 h of nursing by dams injected with either [^14^C]L- or [^14^C]D-BMAA (3.5 h after administration). The levels of [^14^C]L- and [^14^C]D-BMAA in stomach milk seemed to reach a peak after 8 h of nursing (Fig. 3A), but remained high after 24 h of nursing (Figs. 3B and C). In pups killed after 24 h of nursing, a marked and selective uptake of radioactivity was found in tissues with a high protein synthesis and cell turnover such as the intestinal mucosa, bone marrow, thymus and spleen. The uptake of [^14^C]D-BMAA in these tissues was less pronounced than that of [^14^C]L-BMAA. The highest levels of radioactivity in the brain and spinal cord occurred at 24 h after administration of either enantiomer. Analogous to a previous study (sc injections of 10-day-old mouse pups with [^3^H]L-BMAA [21]), pups from dams exposed to [^14^C]L-BMAA revealed a selective distribution of radioactivity in discrete regions of the brain such as the striatum, hippocampus, cerebellum, brainstem and spinal cord at all survival intervals. Pups from dams exposed to [^14^C]D-BMAA showed a slower and less pronounced uptake in these regions than those exposed of [^14^C]L-BMAA. The levels of [^14^C]L- and [^14^C]D-BMAA in the circulating blood remained low at all time-points (Figs. 3B and C).

### Secretion into milk and uptake of [^14^C]L- and [^14^C]D-BMAA in suckling pups

**Figure 4:**
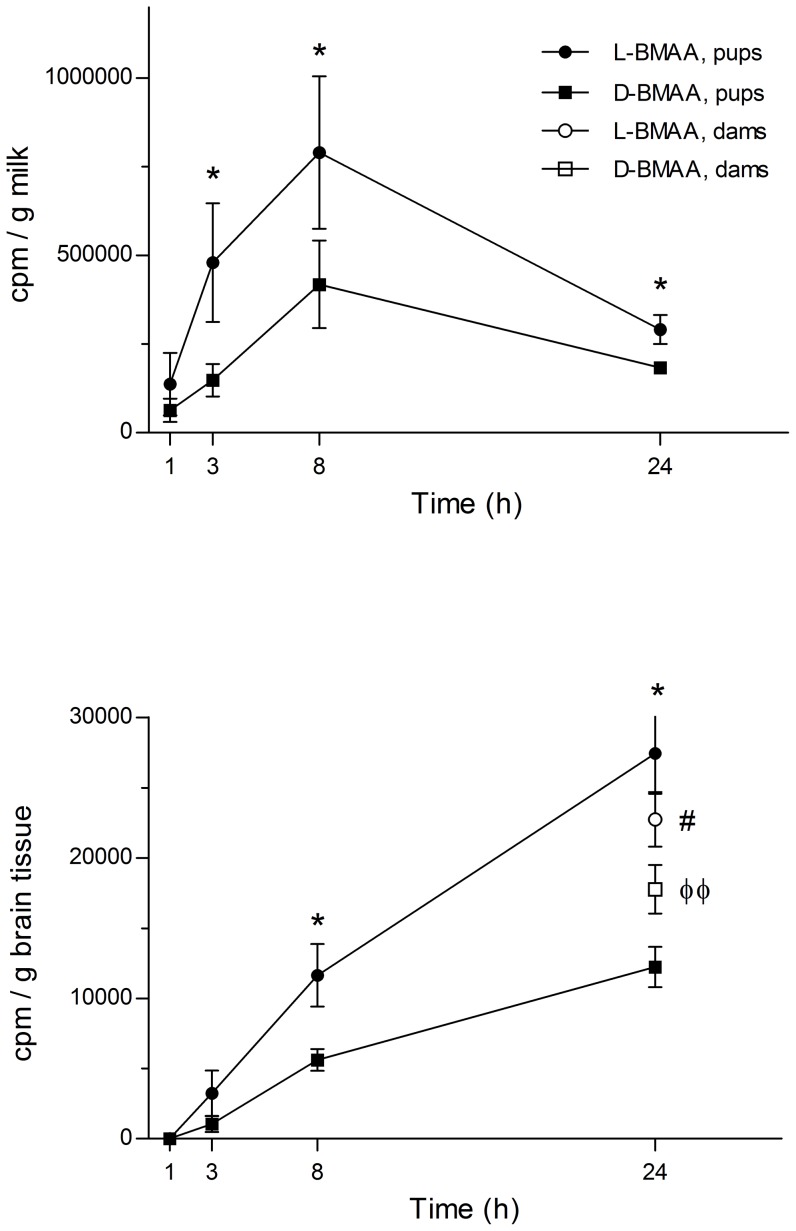
Levels of [^14^C]-labeled L- and D-BMAA in pup stomach milk and brain following administration to the nursing dams. **A** Levels of radioactivity in coagulated stomach milk in suckling mouse pups nursed by dams given a single injection of [^14^C]L- and [^14^C]D-BMAA. The level of L-BMAA in the milk is significantly higher than D-BMAA suggesting a high secretion of the L-enantiomer into milk. **B** Levels of radioactivity in the brain of suckling mouse pups nursed by dams given a single injection of [^14^C]L- and [^14^C]D-BMAA. The level of L-BMAA in the brain of the pups is significantly higher compared with D-BMAA. At 24 h there were significantly higher levels of L-BMAA in the brains of suckling pups compared to the levels in the maternal brains. At the end of the experiment the levels of L-BMAA in the maternal brains were significantly higher than the levels of D-BMAA. Mean values (cpm/g coagulated milk or tissue) ± SD are plotted. *p<0.05 compared to D-BMAA within each time point, #*p*<0.05 compared to their pups, and φ φ *p*<0.01 compared to dams treated with L-BMAA (Mann-Whitney *U*-test). The data analysis is based on the dam as a statistical unit (2 pups/dam/time point).

Both [^14^C]L-BMAA and [^14^C]D-BMAA were transferred via the milk to the suckling pups. As shown in Figure 4A, the level of [^14^C]L-BMAA in coagulated stomach milk was significantly higher than the level of the D-enantiomer at all time-points examined. The levels of both [^14^C]L-, and [^14^C]D-BMAA peaked at about 8 h after single exposure of the dams but there was still high levels in the stomach milk at the last time-point studied, 24 h.

As shown in Figure 4B, there was also a continuous transfer of both labeled enantiomers to the neonatal brain during the investigative period, and there was twofold higher levels of [^14^C]L-BMAA in the neonatal brain as compared to [^14^C]D-BMAA. At the last time point measured there were significantly higher levels of [^14^C]L-BMAA in the brains of suckling pups compared to the levels in the maternal brains (17% higher, p<0.05). In contrast, there were lower levels of [^14^C]D-BMAA in the brains of pups compared to the levels in the maternal brains (45% lower, p = 0.057). The levels of [^14^C]L-BMAA in the maternal brains were significantly higher than the levels of [^14^C]D-BMAA (28%, p<0.01) (Fig. 4B).

### Influx of [^14^C]L- and [^14^C]D-BMAA in mammary HC11 cells

**Figure 5:**
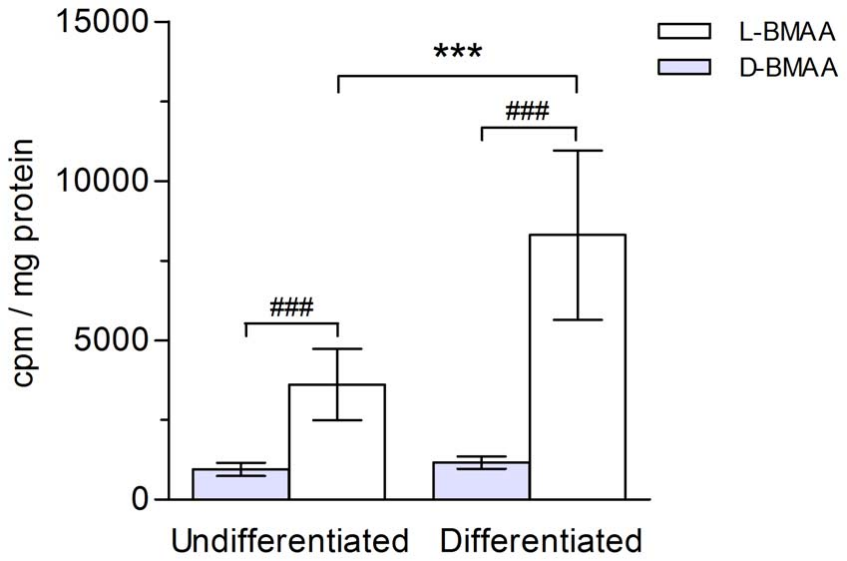
Influx of [^14^C]-labeled L- and D-BMAA in mammary gland HC11 cells. Levels of radioactivity in cultured undifferentiated and differentiated mammary gland cells exposed to [^14^C]-labeled BMAA (1 µM) during 15 min. [^14^C]L-BMAA is taken up at a significantly higher rate than [^14^C]D-BMAA in both undifferentiated and differentiated cells. Differentiation of the mammary gland cells to a secretory state significantly increases the uptake of [^14^C]L- but not [^14^C]D-BMAA. Mean values (cpm/mg protein) from seven experiments ± SD are plotted. ***p<0.001 compared to undifferentiated cells, and ^###^ *p*<0.001 compared to D-BMAA within same cell phenotype.

In order to mimic the physiological status of lactating mammary epithelial cells in vivo, the cell line HC11 was differentiated into a casein-producing phenotype by the use of prolactine. In a first experiment, the uptake of [^14^C]L-BMAA and [^14^C]D-BMAA was measured and compared in non-differentiated and differentiated HC11 cells. As shown in Figure 5, the uptake of [^14^C]L-BMAA during 15 min was increased about 2.5-fold in differentiated cells, compared to undifferentiated cells. For [^14^C]D-BMAA there was no difference in uptake in differentiated and undifferentiated cells. The uptake of the L-enantiomer in differentiated cells was seven times higher than that of the D-enantiomer, while in undifferentiated cells the uptake of [^14^C]L-BMAA was 4 times higher than that of [^14^C]D-BMAA (Fig. 5). These data suggested that differentiated HC11 cells could serve as a suitable in vitro model to examine the transport of BMAA over cell membranes. All further experiments were therefore performed using differentiated cells.

**Figure 6:**
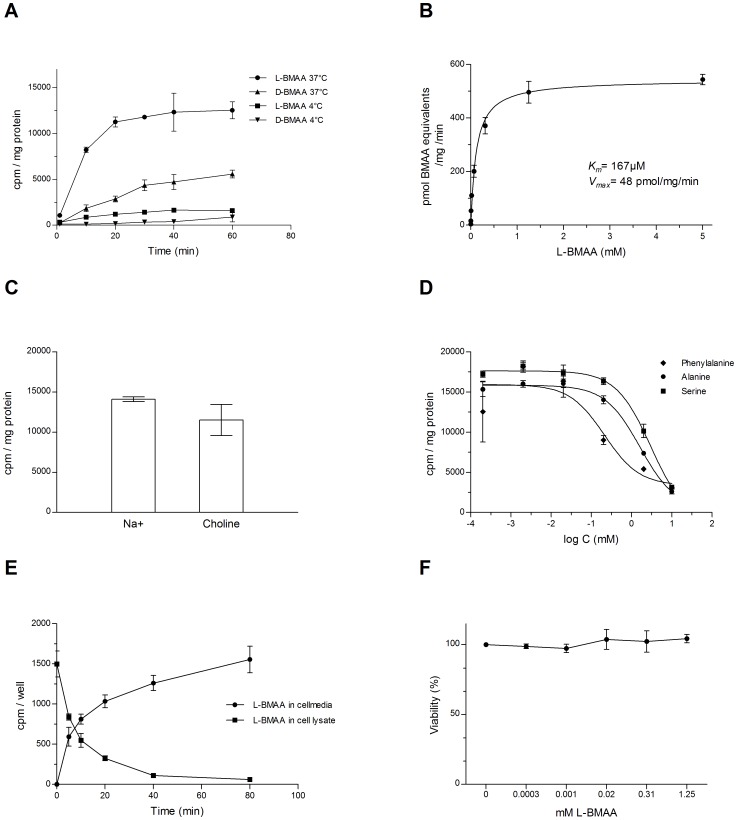
Transport of L- and D-BMAA in differentiated mammary gland HC11 cells. **A** Influx of 1 µM [^14^C]L-BMAA or [^14^C]D-BMAA up to 60 min exposure in cultured mammary gland cells. The influx was highly reduced at incubation at 4°C, compared to incubation at 37°C. **B** Concentration dependent influx of [^14^C]L-BMAA in the presence of unlabeled L-BMAA (0.0003–5 mM) during 15 min exposure in cultured mammary gland cells. The influx of L-BMAA was saturable. **C** Influx of 1 µM [^14^C]L-BMAA or [^14^C]D-BMAA during 15 min exposure in cultured mammary gland cells. Replacement of sodium ions with 120 mM choline chloride did not have a major impact on the influx of [^14^C]L-BMAA. **D** Influx of 1 µM [^14^C]L-BMAA or [^14^C]D-BMAA during 15 min exposure in cultured mammary gland cells. The presence of competing amino acids reduced the influx of [^14^C]L-BMAA. **E** Efflux of 1 µM [^14^C]L-BMAA up to 80 min from cultured preloaded mammary gland cells. A time-dependent efflux is illustrated by decreasing concentrations in the cells and increasing concentrations in the cell medium. Representative experiments are shown in A-E. **F** Viability of mammary cells exposed to increasing concentrations of L-BMAA for 3 h, as determined by MTT assay. Data are presented as mean values (% of vehicle) ± SD (n = 3-4/data-point).

In a following experiment, the time-course for the uptake of BMAA into differentiated HC11 cells was examined during 1 h. As shown in Figure 6A, the uptake process for [^14^C]L-BMAA was linear for about 20 min at 37°C, after which the rate of uptake leveled off. The rate of uptake of [^14^C]D-BMAA was lower than that of [^14^C]L-BMAA and remained linear for roughly 30 min. When measured at 4°C, the rate of uptake of both enantiomers was strongly reduced. Also at 4°C, the rate of uptake of [^14^C]L-BMAA was higher than that of [^14^C]D-BMAA, at least during the early time-points. Based on these results, all further experiments were carried out following 15 min of incubation.

To determine the capacity of the influx mechanism, the uptake of [^14^C]L-BMAA in HC11 cells was examined at various concentrations ranging from 0.3 µM–5 mM. By fitting the data sets from four separate experiments to a Michaelis-Menten equation, it was shown that L-BMAA was transported over the cell membrane by a system that is saturable, resulting in an apparent *K_m_* of 167 µM and a *V_max_* of 48 pmol BMAA equivalents/mg/minute (Fig. 6B).

Displacement of sodium ions with choline chloride did not affect the uptake of [^14^C]L-BMAA to a high degree, even though a slight decrease in uptake could be seen indicating a predominant sodium-independent transport (Fig. 6C).

The uptake of [^14^C]L-BMAA into differentiated HC11 cells was inhibited in a dose dependent fashion by the presence of competing amino acids such as phenylalanine, alanine and serine, with the former being most efficient in inhibiting the influx of [^14^C]L-BMAA (Fig. 6D).

### Efflux of [^14^C]L- and [^14^C]D-BMAA from preloaded HC11 cells

As shown above, the uptake studies revealed that differentiated HC11 cells have a pronounced ability to accumulate the hydrophilic amino acid BMAA (a zwitter ion) from the surrounding medium. A similar uptake mechanism is likely to be in operation in vivo. Another step in the secretion of BMAA into milk would therefore be to transfer the amino acid over the cell membrane of the mammary epithelium into the milk ducts, either as an amino acid or incorporated into milk proteins. To mimic this process, HC11 cells were loaded with [^14^C]L-BMAA from the medium for 40 min. When BMAA-loaded cells were transferred to fresh medium, the cells displayed an ability to secrete [^14^C]L-BMAA into the medium, while the concentration of [^14^C]L-BMAA in the cells was subsequently decreased (Fig. 6E). This efflux process was only shown for the L-enantiomer at 37°C, while no efflux of [^14^C]D-BMAA could be recorded at the conditions employed (data not shown). Likewise, following uptake at 37°C and incubation at 4°C, no efflux of either BMAA enantiomer from the cells to the surrounding medium could be observed (data not shown).

### Effects of L-BMAA on the viability of mammary HC11 cells

No effects of L-BMAA (0.3 µM – 1.25 mM) on the viability of differentiated mammary HC11 cells could be observed during the exposure conditions employed (Fig. 6F).

## Discussion

The neonatal period in rodents is a particularly sensitive period for exposure to neurotoxins. Long-term cognitive disturbances, biochemical and morphological brain changes in adult rodents have been observed after neonatal exposure to the cyanobacterial amino acid and neurotoxin BMAA. Interestingly, the results of the present study revealed that secretion of BMAA into milk is an important route of elimination of L-BMAA in lactating mice resulting in a substantial exposure of the suckling neonatal pups. This conclusion is also supported by the observed high retention of [^14^C]L-BMAA in the tissues of the dam following disrupted nursing. In the suckling pups, the highest concentration of L-BMAA-derived radioactivity were found in the stomach milk at all time-points examined (3–24 h). The concentrations in the brains of the suckling pups increased during the whole studied time period. Notably, at 24 h the levels of L-BMAA in the neonatal brains significantly exceeded the levels in the maternal brains. A continued nursing period beyond 24 h would thus likely result in even higher L-BMAA-concentrations in the neonatal brain than in the adult brain, as was previously demonstrated in fetal mice exposed over the placenta [18]. We have recently reported that L-BMAA is a developmental neurotoxicant that induces persistent and dose-dependent behavioral, proteomic and degenerative changes in specific brain regions in adult or postnatal rodents exposed subcutaneously to BMAA during postnatal day 9-10, i.e. at a critical phase of brain development [21]–[25]. The corresponding period in humans starts during the last trimester of pregnancy and lasts for the first few years of age [27]. The current results are therefore of particular interest for human risk assessment, mainly because they raise the possibility that exposures of breast-fed babies to BMAA could occur. The results further imply that cow's milk could be an additional source of human exposure to BMAA besides for instance fish and shellfish [12], [13], [15]. Despite the findings that BMAA is formed by a variety of aquatic and terrestrial cyanobacterial species and biomagnified in aquatic and terrestrial food webs, the magnitude of human exposure remains largely unknown. Consequently, there is an urgent need to determine whether the developmental cyanobacterial neurotoxin BMAA may be enriched in mother's milk and cow's milk, and other dairy consumer products.

In addition to being a neutral zwitter ion, the amino acid BMAA can appear also as a tripolar cation at physiological pH. Moreover, BMAA forms tripolar carbamate ions at physiological HCO_3_ concentrations [39], [40]. The uptake of BMAA into the mammary gland and the subsequent secretion into milk should therefore not be expected to occur by passive diffusion but rather be mediated by specific transport mechanisms, as has previously been implicated for the transfer of L-BMAA over the blood-brain barrier in rodents [17] and influx to the neuroblastoma cell line SH-SY5Y [41] where the large amino acid transporter (LAT1) were suggested to be involved.

The mammary gland expresses several amino acid transporters, including the sodium-dependent system A and the sodium-independent system L. System A transports small, neutral and N-methylated amino acids, e.g. alanine and serine while system L, which includes LAT1 and LAT2, transports large, neutral, branched and aromatic amino acids with low stereospecificity, e.g. phenylalanine [42]–[44]. A major distinction between LAT1 and 2 is that LAT2 has a wider substrate specificity than LAT1, and unlike LAT1, transports L-alanine [45], [46].

The mouse mammary epithelial HC11 cell line was employed as a model to examine the influx and efflux of L- and D-BMAA in the mammary epithelium in vitro. The level of influx of L-BMAA into differentiated cells was seven times higher than that of D-BMAA, suggesting an enantiomer-selective uptake. Accordingly, the level of influx of L-BMAA increased when the cells were differentiated into a casein-producing phenotype, while the influx of D-BMAA was not altered. In addition, the uptake of L-BMAA increased exponentially to a maximal level within 20 min, while the uptake curve for D-BMAA was lower and more flat. These results suggest that the putative L-BMAA transporter was up-regulated by differentiating the HC11 cells into a secretory state; they also conform with the observation that the uptake of ^14^C-labeled L-BMAA in the mammary gland of non-pregnant nulliparous mice was very low compared to that of lactating mice (unpublished data). We therefore conclude that the L-enantiomer was favored over the D-enantiomer by the putative BMAA transporter although we cannot rule out that there was a transport also of the D-enantiomer. Other D-amino acids have previously been reported to be transported by common transporters; for instance, D-aspartic acid is transported via the same system as L-aspartic and L-glutamic acid [47]. Notably, the difference in cellular uptake between the L- and D-BMAA enantiomers in the HC11 cell line in vitro was more pronounced than the differences in milk secretion and uptake in the neonatal brain of the two enantiomers in vivo. These observations support the intriguing possibility that racemization [48], [49] of D-BMAA into L-BMAA, or vice versa, may take place. If this indeed was the case, racemization should be expected to be more efficient in the physiologically complex in vivo system than in HC11 cells vitro.

The rapid and saturable influx of L-BMAA into differentiated HC11 cells revealed an apparent *K_m_* value of 167 µM and *V_max_* value of 48 pmol/mg protein/min. These values were similar to those recorded for other amino acids in vitro, e.g. cultures of astrocytes and C6 glioma cells has a reported *K_m_* of 224 µM and *V_max_* of 52 µM respectively for L-leucine [50]. The competition experiments further revealed that L-BMAA influx was inhibited both by L-phenylalanine, L- alanine and L- serine, the former amino acid being the most efficient competitor with an apparent IC_50_ of about 200 µM. Based on these data and the seemingly low sodium-dependency of the cellular uptake, we suggest that LAT1 and LAT2 were involved in the transport of BMAA. Further studies are, however, needed to characterize the transporters regulating the influx and efflux of L-BMAA in mammary gland epithelial cells.

The majority of amino acids recruited to the mammary gland are rapidly used for synthesis of milk proteins, e.g. casein, which are secreted via secretory vesicles into the alveolar lumen [51], [52]. Yet, approximately 5% are still present as free amino acids in milk and have been suggested to play a beneficial role during early postnatal development [53], [54]. The efflux of radioactivity from HC11 cells preloaded with [^14^C]L-BMAA was almost complete within 80 min. Consequently, there was also a rapid and efficient efflux mechanism for L-BMAA in the HC11 cell line. Further studies are required to demonstrate whether the efflux of radioactivity represents free L-BMAA or L-BMAA associated/incorporated to/into milk proteins such as casein. BMAA has previously been shown to be bound to cycad seed flour protein and such an association should be a prerequisite for biomagnification of BMAA in the food chain, leading to high levels of BMAA in flying foxes, shark fins, and in the brain and other tissues in fish [6], [12], [13], [15].

Due to the proposed role of BMAA in the etiology of neurodegenerative disease, the search for BMAA in humans and other species has been focused on the central nervous system [2], [55]. It is therefore notable that BMAA showed a typical amino acid-like tissue distribution pattern with the highest levels in tissues with a high cell proliferation and/or protein synthesis, i.e. in the thymus and spleen, bone marrow, gastrointestinal mucosa and the pancreas. These findings are compatible with the hypothesis that BMAA may be incorporated as a false amino acid into proteins [56], and that this incorporation could lead to protein misfolding, a hallmark of neurodegenerative disease [57]. In this context it is interesting to note that the uptake of BMAA in the growing fetal and neonatal brain, with high protein synthesis, becomes higher than that of the adult brain in pregnant [21] and lactating mice. The targeting of BMAA to the developing brain and to tissues with a high cell proliferation and protein synthesis could also result from distinct expression of the putative amino acid transporter(s) in these tissues. Experiments are in progress to clarify these possibilities.

## Conclusions

We have shown for the first time that secretion of BMAA into milk is an efficient exposure pathway in suckling neonatal mice. Following secretion of L-BMAA into milk, the levels of L-BMAA in the neonatal brain significantly exceeded the levels in the maternal brain following 24 h of nursing. The influx of BMAA in cultured mouse mammary epithelial cells was saturable and seemed to be dependent on amino acid transporters, most likely LAT1 and LAT2. Given the persistent neurodevelopmental toxicity recently described following injection of L-BMAA to neonatal rodent pups, the current results highlight the need to determine whether BMAA is enriched also in human milk and in cow's milk. In addition to highlighting the exposure of nursed babies, the presence of BMAA in mother's milk could possibly become a biomarker for exposure and body-burden of BMAA in adults. The HC11 cell line appears to be an attractive in vitro system to further characterize the mechanism of secretion of BMAA into milk.

## References

[1] Spencer PS, Hugon J, Ludolph A, Nunn PB, Ross SM, et al (1987) Discovery and partial characterization of primate motor-system toxins. Ciba Found Symp 126: 221–238. 3107939 10.1002/9780470513422.ch14

[2] Cox PA, Banack SA, Murch SJ (2003) Biomagnification of cyanobacterial neurotoxins and neurodegenerative disease among the Chamorro people of Guam. Proc Natl Acad Sci U S A 100: 13380–13383. 14612559 10.1073/pnas.2235808100 PMC263822

[3] Murch SJ, Cox PA, Banack SA, Steele JC, Sacks OW (2004) Occurrence of beta-methylamino-l-alanine (BMAA) in ALS/PDC patients from Guam. Acta Neurol Scand 110: 267–269. 15355492 10.1111/j.1600-0404.2004.00320.x

[4] Pablo J, Banack SA, Cox PA, Johnson TE, Papapetropoulos S, et al (2009) Cyanobacterial neurotoxin BMAA in ALS and Alzheimer's disease. Acta Neurol Scand 120: 216–225. 19254284 10.1111/j.1600-0404.2008.01150.x

[5] Bradley WG, Borenstein AR, Nelson LM, Codd GA, Rosen BH, et al (2013) Is exposure to cyanobacteria an environmental risk factor for amyotrophic lateral sclerosis and other neurodegenerative diseases? Amyotroph Lateral Scler Frontotemporal Degener 14: 325–333. 23286757 10.3109/21678421.2012.750364

[6] Banack SA, Cox PA (2003) Biomagnification of cycad neurotoxins in flying foxes: implications for ALS-PDC in Guam. Neurology 61: 387–389. 12913204 10.1212/01.wnl.0000078320.18564.9f

[7] Spencer PS, Nunn PB, Hugon J, Ludolph AC, Ross SM, et al (1987) Guam amyotrophic lateral sclerosis-parkinsonism-dementia linked to a plant excitant neurotoxin. Science 237: 517–522. 3603037 10.1126/science.3603037

[8] Banack SA, Johnson HE, Cheng R, Cox PA (2007) Production of the neurotoxin BMAA by a marine cyanobacterium. Mar Drugs 5: 180–196. 18463731 10.3390/md504180 PMC2365698

[9] Cox PA, Banack SA, Murch SJ, Rasmussen U, Tien G, et al (2005) Diverse taxa of cyanobacteria produce beta-N-methylamino-L-alanine, a neurotoxic amino acid. Proc Natl Acad Sci U S A 102: 5074–5078. 15809446 10.1073/pnas.0501526102 PMC555964

[10] Metcalf JS, Richer R, Cox PA, Codd GA (2012) Cyanotoxins in desert environments may present a risk to human health. Sci Total Environ 421–422: 118–123. 10.1016/j.scitotenv.2012.01.053 22369867

[11] Prasanna R, Joshi M, Rana A, Shivay YS, Nain L (2012) Influence of co-inoculation of bacteria-cyanobacteria on crop yield and C-N sequestration in soil under rice crop. World J Microbiol Biotechnol 28: 1223–1235. 22805842 10.1007/s11274-011-0926-9

[12] Brand LE, Pablo J, Compton A, Hammerschlag N, Mash DC (2010) Cyanobacterial Blooms and the Occurrence of the neurotoxin beta-N-methylamino-L-alanine (BMAA) in South Florida Aquatic Food Webs. Harmful Algae 9: 620–635. 21057660 10.1016/j.hal.2010.05.002 PMC2968748

[13] Jonasson S, Eriksson J, Berntzon L, Spacil Z, Ilag LL, et al (2010) Transfer of a cyanobacterial neurotoxin within a temperate aquatic ecosystem suggests pathways for human exposure. Proc Natl Acad Sci U S A 107: 9252–9257. 20439734 10.1073/pnas.0914417107 PMC2889067

[14] Metcalf JS, Banack SA, Kotut K, Krienitz L, Codd GA (2013) Amino acid neurotoxins in feathers of the Lesser Flamingo, Phoeniconaias minor. Chemosphere 90: 835–839. 23123117 10.1016/j.chemosphere.2012.09.094

[15] Mondo K, Hammerschlag N, Basile M, Pablo J, Banack SA, et al (2012) Cyanobacterial neurotoxin beta-N-methylamino-L-alanine (BMAA) in shark fins. Mar Drugs 10: 509–520. 22412816 10.3390/md10020509 PMC3297012

[16] Field NC, Metcalf JS, Caller TA, Banack SA, Cox PA, et al (2013) Linking beta-methylamino-L-alanine exposure to sporadic amyotrophic lateral sclerosis in Annapolis, MD. Toxicon 70: 179–183. 23660330 10.1016/j.toxicon.2013.04.010

[17] Smith QR, Nagura H, Takada Y, Duncan MW (1992) Facilitated transport of the neurotoxin, beta-N-methylamino-L-alanine, across the blood-brain barrier. J Neurochem 58: 1330–1337. 1548467 10.1111/j.1471-4159.1992.tb11346.x

[18] Karlsson O, Berg C, Brittebo EB, Lindquist NG (2009a) Retention of the cyanobacterial neurotoxin beta-N-methylamino-l-alanine in melanin and neuromelanin-containing cells—a possible link between Parkinson-dementia complex and pigmentary retinopathy. Pigment Cell Melanoma Res 22: 120–130. 19154235 10.1111/j.1755-148X.2008.00508.x

[19] Xie X, Basile M, Mash DC (2013) Cerebral uptake and protein incorporation of cyanobacterial toxin beta-N-methylamino-L-alanine. Neuroreport 24: 779–784. 23979257 10.1097/WNR.0b013e328363fd89

[20] Perry TL, Bergeron C, Biro AJ, Hansen S (1989) Beta-N-methylamino-L-alanine. Chronic oral administration is not neurotoxic to mice. J Neurol Sci 94: 173–180. 2614465 10.1016/0022-510x(89)90227-x

[21] Karlsson O, Lindquist NG, Brittebo EB, Roman E (2009b) Selective brain uptake and behavioral effects of the cyanobacterial toxin BMAA (beta-N-methylamino-L-alanine) following neonatal administration to rodents. Toxicol Sci 109: 286–295. 19321797 10.1093/toxsci/kfp062

[22] Karlsson O, Berg AL, Lindstrom AK, Hanrieder J, Arnerup G, et al (2012) Neonatal exposure to the cyanobacterial toxin BMAA induces changes in protein expression and neurodegeneration in adult hippocampus. Toxicol Sci 130: 391–404. 22872059 10.1093/toxsci/kfs241 PMC3498744

[23] Karlsson O, Roman E, Berg AL, Brittebo EB (2011) Early hippocampal cell death, and late learning and memory deficits in rats exposed to the environmental toxin BMAA (beta-N-methylamino-L-alanine) during the neonatal period. Behav Brain Res 219: 310–320. 21315110 10.1016/j.bbr.2011.01.056

[24] Karlsson O, Roman E, Brittebo EB (2009c) Long-term cognitive impairments in adult rats treated neonatally with beta-N-Methylamino-L-Alanine. Toxicol Sci 112: 185–195. 19692667 10.1093/toxsci/kfp196

[25] Karlsson O, Kultima K, Wadensten H, Nilsson A, Roman E, et al. (2013) Neurotoxin-Induced Neuropeptide Perturbations in Striatum of Neonatal Rats. J Proteome Res. 10.1021/pr3010265 23410195

[26] Engskog MK, Karlsson O, Haglof J, Elmsjo A, Brittebo E, et al. (2013) The cyanobacterial amino acid beta-N-methylamino-l-alanine perturbs the intermediary metabolism in neonatal rats. Toxicology. 10.1016/j.tox.2013.07.010 23886855

[27] Dobbing J, Sands J (1979) Comparative aspects of the brain growth spurt. Early Hum Dev 3: 79–83. 118862 10.1016/0378-3782(79)90022-7

[28] Eriksson P (1997) Developmental neurotoxicity of environmental agents in the neonate. Neurotoxicology 18: 719–726. 9339819

[29] Duncan MW, Markey SP, Weick BG, Pearson PG, Ziffer H, et al (1992) 2-Amino-3-(methylamino)propanoic acid (BMAA) bioavailability in the primate. Neurobiol Aging 13: 333–337. 1522948 10.1016/0197-4580(92)90047-2

[30] Duncan MW, Villacreses NE, Pearson PG, Wyatt L, Rapoport SI, et al (1991) 2-amino-3-(methylamino)-propanoic acid (BMAA) pharmacokinetics and blood-brain barrier permeability in the rat. J Pharmacol Exp Ther 258: 27–35. 2072299

[31] Ullberg S (1977) The technique of whole body autoradiography, cryosectioning of large specimens. Sci Tools LKB Instr J: 2–29.

[32] Ball RK, Friis RR, Schoenenberger CA, Doppler W, Groner B (1988) Prolactin regulation of beta-casein gene expression and of a cytosolic 120-kd protein in a cloned mouse mammary epithelial cell line. EMBO J 7: 2089–2095. 3416834 10.1002/j.1460-2075.1988.tb03048.x PMC454494

[33] Jäger (2008) Lactogenic differentiation of HC11 cells is not accompanied by downregulation of AP-2 transcription factor genes. BMC reseach notes 1 10.1186/1756-0500-1-29 PMC2518283 18710545

[34] Malathi P, Ramaswamy K, Caspary WF, Crane RK (1973) Studies on the transport of glucose from disaccharides by hamster small intestine in vitro. I. Evidence for a disaccharidase-related transport system. Biochim Biophys Acta 307: 613–626. 4718809 10.1016/0005-2736(73)90306-4

[35] Pineda M, Fernandez E, Torrents D, Estevez R, Lopez C, et al (1999) Identification of a membrane protein, LAT-2, that Co-expresses with 4F2 heavy chain, an L-type amino acid transport activity with broad specificity for small and large zwitterionic amino acids. J Biol Chem 274: 19738–19744. 10391915 10.1074/jbc.274.28.19738

[36] Uchino H, Kanai Y, Kim DK, Wempe MF, Chairoungdua A, et al (2002) Transport of amino acid-related compounds mediated by L-type amino acid transporter 1 (LAT1): insights into the mechanisms of substrate recognition. Mol Pharmacol 61: 729–737. 11901210 10.1124/mol.61.4.729

[37] Zerangue N, Kavanaugh MP (1996) ASCT-1 is a neutral amino acid exchanger with chloride channel activity. J Biol Chem 271: 27991–27994. 8910405 10.1074/jbc.271.45.27991

[38] Tada H, Shiho O, Kuroshima K, Koyama M, Tsukamoto K (1986) An improved colorimetric assay for interleukin 2. J Immunol Methods 93: 157–165. 3490518 10.1016/0022-1759(86)90183-3

[39] Nunn PB, O'Brien P (1989) The interaction of beta-N-methylamino-L-alanine with bicarbonate: an 1H-NMR study. FEBS Lett 251: 31–35. 2666171 10.1016/0014-5793(89)81423-1

[40] Myers TG, Nelson SD (1990) Neuroactive carbamate adducts of beta-N-methylamino-L-alanine and ethylenediamine. Detection and quantitation under physiological conditions by 13C NMR. J Biol Chem 265: 10193–10195. 2113048

[41] Okle O, Stemmer K, Deschl U, Dietrich DR (2013) L-BMAA induced ER stress and enhanced caspase 12 cleavage in human neuroblastoma SH-SY5Y cells at low nonexcitotoxic concentrations. Toxicol Sci 131: 217–224. 23047912 10.1093/toxsci/kfs291

[42] Verma N, Kansal VK (1993) Characterisation of the routes of methionine transport in mouse mammary glands. Indian J Med Res 98: 297–304. 8132234

[43] Shennan DB, Calvert DT, Travers MT, Kudo Y, Boyd CA (2002) A study of L-leucine, L-phenylalanine and L-alanine transport in the perfused rat mammary gland: possible involvement of LAT1 and LAT2. Biochim Biophys Acta 1564: 133–139. 12101005 10.1016/s0005-2736(02)00410-8

[44] Aleman G, Lopez A, Ordaz G, Torres N, Tovar AR (2009) Changes in messenger RNA abundance of amino acid transporters in rat mammary gland during pregnancy, lactation, and weaning. Metabolism 58: 594–601. 19375580 10.1016/j.metabol.2008.12.003

[45] Rajan DP, Kekuda R, Huang W, Devoe LD, Leibach FH, et al (2000) Cloning and functional characterization of a Na(+)-independent, broad-specific neutral amino acid transporter from mammalian intestine. Biochim Biophys Acta 1463: 6–14. 10631289 10.1016/s0005-2736(99)00224-2

[46] Rossier G, Meier C, Bauch C, Summa V, Sordat B, et al (1999) LAT2, a new basolateral 4F2hc/CD98-associated amino acid transporter of kidney and intestine. J Biol Chem 274: 34948–34954. 10574970 10.1074/jbc.274.49.34948

[47] Drejer J, Larsson OM, Schousboe A (1983) Characterization of uptake and release processes for D- and L-aspartate in primary cultures of astrocytes and cerebellar granule cells. Neurochem Res 8: 231–243. 6856028 10.1007/BF00963923

[48] Hasegawa H, Matsukawa T, Shinohara Y, Konno R, Hashimoto T (2004) Role of renal D-amino-acid oxidase in pharmacokinetics of D-leucine. Am J Physiol Endocrinol Metab 287: E160–165. 15026304 10.1152/ajpendo.00397.2003

[49] Wolosker H, Sheth KN, Takahashi M, Mothet JP, Brady RO Jr, et al (1999) Purification of serine racemase: biosynthesis of the neuromodulator D-serine. Proc Natl Acad Sci U S A 96: 721–725. 9892700 10.1073/pnas.96.2.721 PMC15203

[50] Kim DK, Kim IJ, Hwang S, Kook JH, Lee MC, et al (2004) System L-amino acid transporters are differently expressed in rat astrocyte and C6 glioma cells. Neurosci Res 50: 437–446. 15567481 10.1016/j.neures.2004.08.003

[51] McManaman JL, Neville MC (2003) Mammary physiology and milk secretion. Adv Drug Deliv Rev 55: 629–641. 12706546 10.1016/s0169-409x(03)00033-4

[52] Shennan DB, Peaker M (2000) Transport of milk constituents by the mammary gland. Physiol Rev 80: 925–951. 10893427 10.1152/physrev.2000.80.3.925

[53] Sarwar G, Botting HG, Davis TA, Darling P, Pencharz PB (1998) Free amino acids in milks of human subjects, other primates and non-primates. Br J Nutr 79: 129–131. 9536856 10.1079/bjn19980023

[54] Svanberg U, Gebre-Medhin M, Ljungqvist B, Olsson M (1977) Breast milk composition in Ethiopian and Swedish mothers. III. Amino acids and other nitrogenous substances. Am J Clin Nutr 30: 499–507. 557894 10.1093/ajcn/30.4.499

[55] Ross SM, Seelig M, Spencer PS (1987) Specific antagonism of excitotoxic action of ‘uncommon’ amino acids assayed in organotypic mouse cortical cultures. Brain Res 425: 120–127. 3123008 10.1016/0006-8993(87)90490-2

[56] Holtcamp W (2012) The emerging science of BMAA: do cyanobacteria contribute to neurodegenerative disease? Environ Health Perspect 120: A110–116. 22382274 10.1289/ehp.120-a110 PMC3295368

[57] Moreno-Gonzalez I, Soto C (2011) Misfolded protein aggregates: mechanisms, structures and potential for disease transmission. Semin Cell Dev Biol 22: 482–487. 21571086 10.1016/j.semcdb.2011.04.002 PMC3175247

