# Correction: Maternal Transfer of the Cyanobacterial Neurotoxin β-N-Methylamino-L-Alanine (BMAA) via Milk to Suckling Offspring

**DOI:** 10.1371/journal.pone.0133110

**Published:** 2015-07-14

**Authors:** Marie Andersson, Oskar Karlsson, Ulrika Bergström, Eva B. Brittebo, Ingvar Brandt

During the preparation of Michaelis-Menten calculations an erroneous factor was included which resulted in erroneous K_m_- and V_max_ values. The correct apparent K_m_-value for differentiated HC11 cells is 3.1 mM, and the correct apparent V_max_-value is 30.4 nmol/mg protein/min.
